# Early career psychiatrists in europe during COVID-19 outbreak: Results of the EPA ECPC-EFPT cross-sectional survey

**DOI:** 10.1192/j.eurpsy.2021.139

**Published:** 2021-08-13

**Authors:** T. Gondek

**Affiliations:** Early Career Psychiatrists Committee, European Psychiatric Association, Wroclaw, Poland

**Keywords:** education in psychiatry, COVID-19, telemedicine, Early career psychiatrists

## Abstract

The COVID-19 outbreak has left its mark on the work of mental health care staff. Many professionals had to radically change their working conditions or were delegated to work in different facilities, in many cases taking on different responsibilities with little time for training. Many psychiatrists overnight had to partially or fully start working within telemedicine. Due to the lockdown, psychiatric trainees in many countries were not able to complete their training as planned. The measures taken by the governments to limit the impact of the pandemic also affected the capacity to conduct research studies and directions of new research initiatives. Dr. Gondek will present the results of the EPA ECPC-EFPT Cross-sectional survey on the effects of the outbreak on work and wellbeing of Early Career Psychiatrists in Europe.

**Disclosure:**

No significant relationships.

